# Incidence and prevalence of hepatitis C and B infections among men who have sex with men and transgender women enrolled in a United States HIV vaccine trial

**DOI:** 10.21203/rs.3.rs-4474493/v1

**Published:** 2024-06-14

**Authors:** Matthew Scherer, Vijay Nandi, Magdalena E Sobieszczyk, Oliver Laeyendecker, Shelly Karuna, Michele Andrasik, Holly E Janes, Erin E Brown, Hong-Van Tieu

**Affiliations:** Columbia University Vagelos School of Physicians and Surgeons; New York Blood Center; Columbia University Vagelos School of Physicians and Surgeons; Johns Hopkins University School of Medicine; Fred Hutchinson Cancer Center; HIV Vaccine Trials Network; Fred Hutchinson Cancer Center; National Institutes of Health; New York Blood Center

**Keywords:** Hepatitis B infection, Hepatitis C infection, men who have sex with men, transgender women

## Abstract

**Background:**

Rising hepatitis C and B virus (HCV and HBV) rates have been reported in men who have sex with men (MSM) and transgender women (TGW). This study characterizes HCV and HBV infections longitudinally among 2,496 MSM/TGW aged 18–50 years and at risk for HIV acquisition enrolled in an HIV-1 vaccine trial in 18 U.S. cities between 2009–2013.

**Methods:**

Participants completed behavioral surveys, HIV testing, and blood collection over 24 months. Of the 2,397 participants who consented for future testing, 1,792 (74.8%) had available paired stored blood samples at baseline and a later timepoint (Month 24 [N = 999]; if unavailable, M12 [N = 775] or M15 [N = 18]).

**Results:**

Among 1,792 participants, 98.1% were MSM, 0.8% were TGW, and the median age was 30 years (IQR 24, 40). Participants reported a median number of 3 male sex partners (IQR 1,5) within the past 3 months. Condomless insertive anal sex was reported by 55.8% and condomless receptive anal sex by 46.7%.1.3% reported injection drug use. During follow-up, 1.4% reported pre-exposure prophylaxis (PrEP) use. At baseline 11/1792 (0.61%) participants had HCV infection (HCV AB positive, RNA detectable), with all having persistent detectable RNA and chronic HCV infection at follow-up. Phylogenetic analysis showed no clusters of HCV infection. 8 participants had HCV AB positive, RNA undetectable at baseline and follow-up, representing past HCV infection with clearance; only 2 acquired HCV, which cleared over 12–24 months. At baseline, 2 participants (2/1792 = 0.11%) had positive HBsAg, indicating chronic HBV infection. Over 12–24 months, 4 (4/1790, 0.22%) developed HBsAg positivity; these participants had HBcAB positivity at baseline, thereby likely representing reactivation. There were no new HBV infections during follow-up.

**Conclusion:**

Among 1,792 men who have sex with men and transgender women aged 18–50 years and at risk for HIV acquisition enrolled in a U.S. HIV-1 vaccine trial, incident hepatitis C infection rates were extremely low, with no cases of incident hepatitis B infection. These rates of incident HCV infection and HBSAg positivity are lower than previously reported among MSM/TGW.

## Introduction

Hepatitis B virus (HBV) and hepatitis C virus (HCV) infections among men who have sex with men (MSM) and transgender women (TGW) are a public health concern of global importance. HCV is transmitted primarily through the parenteral route; however, outbreaks of sexually transmitted HCV infection have been reported among MSM in Europe, Australia, Asia, and North America.^1,2^ These sexually transmitted HCV epidemics have disproportionately affected MSM living with HIV.^3,4^ Risk for HCV acquisition is associated with sexual risk behaviors, including high number of sexual partners, condomless receptive anal sex, recreational drug use during sex, and traumatic sexual practices (e.g., sting, use of sex toys).^1,5–8^ Studies have reported varying incidence and prevalence of HCV in HIV-negative MSM.^3,9–11^ In addition to HCV, MSM and TGW are at higher risk for HBV infection given the increased HBV transmission risk associated with condomless anal sex and reports of relatively high rates of HBV infection among MSM and TGW.^12,13^ This study characterizes baseline and incident HCV and HBV infections among MSM/TGW enrolled in an HIV-1 vaccine trial that closed in 2013. These data provide an important baseline of HBV and HCV incidence and prevalence in this high-risk population that pre-dates the increased availability of HIV pre-exposure prophylaxis (PrEP).

## Materials and Methods

This study used secondary data and stored specimens from HIV Vaccine Trials Network (HVTN) 505, a Phase 2b randomized placebo-controlled trial of a DNA/recombinant adenovirus 5 HIV-1 vaccine.^14^ HVTN 505 enrolled 2,496 MSM/TGW aged 18–50 years and considered at risk for HIV acquisition in 21 U.S. sites between 2009–2013. Participants completed behavioral surveys and underwent HIV testing and blood collection over 24 months. Following the iPrEX study results,^15^ the trial was modified to account for uptake of HIV PrEP, and participants were educated about and provided access to this prevention modality. Study vaccinations were halted early in 2013 for lack of efficacy in preventing HIV acquisition. In the modified intention-to-treat population, yearly HIV incidence was similar in the vaccine [2.7% (95% confidence interval (CI), 1.9 to 3.6)] and placebo [2.1% (95% CI, 1.4 to 2.9)] groups. In subsequent trial modifications, participants were unblinded and followed to 48 months post-enrollment. Of 2,397 participants who consented for future testing, 1,792 (74.8%) had available paired stored blood samples at baseline (month 0) and a later timepoint. Month 24 data was used for 999 participants. If month 24 data were unavailable,, month 12 [n = 775] or month 15 [n = 18] data were used. All 1,792 participants with paired stored blood samples were included in the current analysis.

HCV antibody (HCV Ab) testing was performed, using Genedia anti-HCV 3.0 ELISA (Green Cross, Korea), on stored samples from these visits. If HCV Ab was positive at the later time point, the sample and the corresponding baseline sample were tested for HCV ribonucleic acid (RNA) using the Abbott Real Time HCV Amplification Reagent Kit (No. 04J86–90, Des Plaines, IL), with limit of detection of 50 IU/mL. HCV phylogenetics were analyzed on incident and chronic HCV positives, with extraction of HCV RNA and amplification of HCV E1 gene (H77 nucleotides 943 to 1288) for sequencing and phylogenetic analysis.^16^

HBV testing was performed similarly. Testing for hepatitis B surface antibody (HBsAb), hepatitis B core antibody (HBcAb), and hepatitis B surface antigen (HBsAg) was performed using ETI-AB-AUK PLUS, ETI-AB-COREK PLUS, and ETI-MAK-2 PLUS, respectively (DiaSorin, Saluggia, Italy). Any samples positive for HBSAg were tested for HBV DNA viral load, using RealTime HBV Amplification Reagent Kits by Abbott (lower limit of quantitation 10 IU/mL).

HCV infections were classified as follows: (1) chronic (HCV antibody and RNA positive), (2) cleared (HCV antibody positive, RNA negative; this includes those who were successfully treated for HCV infection and those who had spontaneously cleared HCV), (3) other (HCV antibody negative, RNA positive; those with acute HCV infections), and (4) no HCV infection. Incident HCV infections were those HCV Ab negative and RNA negative at baseline who were HCV Ab positive and RNA positive at the later time point. Incident HBV infections were those without HBV infection at baseline and with HBV infection (positive HBsAg, positive HBcAb, negative HBsAb, and positive HBV DNA viral load) at the later timepoint. Descriptive statistics were computed, and analyses were conducted using Microsoft 365 Excel and R version 4.1.1.

The main HVTN 505 study was approved by the institutional review board at the Fred Hutchinson Cancer Research Center, which served as a central institutional review board for 11 sites through agreements with the institutions. At the remaining 10 sites, the study was approved by the local institutional review board. HVTN 505 study participants provided written informed consent for the main study through the study sites involved in HVTN 505 study, and blood samples were collected and stored for study participants who provided consent for other uses of study samples related to HIV, the immune system and other diseases. This sub-study had been submitted to the New York Blood Center IRB, which had determined this project does not meet the definition of human subject research under the purview of the IRB according to federal regulations.

The data, including stored blood specimens, were accessed for research purposes on October 28, 2019; the authors did not have access to information that could identify individual participants during or after data collection.

## Results

Among the 1,792 participants with available paired blood samples, 98.1% self-identified as MSM and 0.8% as TGW, representing 2,796 person-years of observation. The median age was 30 years (IQR 24, 40); 69.8% identified as White, 15.8% as Black/African American, and 7.3% as Hispanic.

In the prior 3 months, the median number of male sex partners was 3 (IQR 1, 5). Condomless insertive anal sex was reported by 55.8%, and condomless receptive anal sex by 46.7%. Only 1.3% reported injection drug use (IDU). Additionally, 24.1% reported the use of poppers/inhaled nitrates, 12.1% snorted/sniffed powder cocaine, 7.2% used 3,4-methylenedioxymethamphetamine (MDMA), 7.5% used non-injected opiates/benzodiazepines, 5.2% used non-injected methamphetamines/amphetamines, and 3.5% smoked crack cocaine. During follow-up, 1.6% reported PrEP use.

At baseline, 11/1792 (0.61%) participants had known chronic HCV infection, with all having persistent detectable HCV RNA at the later time point. Phylogenetic analysis showed no clusters of HCV infection. Eight participants were HCV Ab positive at baseline with undetectable HCV RNA results at both time points, likely representing HCV infection and clearance prior to study enrollment. During the study, 2 participants (0.11%) who were HCV negative at baseline had subsequent positive HCV Ab with negative HCV RNA at the later time point; both were MSM, with one reporting recent IDU. This probably represents HCV acquired during the study with spontaneous clearance.

Regarding HBV infection, 4 participants (4/1,790, 0.22%) were HBsAg positive at the follow-up visit; 2 of these (2/1,792, 0.11%) had baseline positive HBsAg, indicating chronic HBV infection. The other 2, who were HBsAg negative at baseline, had HBcAb positivity at baseline and HBV DNA negativity at baseline, likely representing reactivation. There were no incident HBV infections during follow-up.

## Discussion

In this study we report only 2 cases of incident HCV and no cases of incident HBV at 12–24 months post-enrollment in a large cohort of MSM and TGW enrolled in an HIV vaccine trial from 2009–2013. These data provide an important baseline for HBV and HCV incidence and prevalence among this high risk population prior to the scaling up of HIV PrEP.

Prior estimates of HCV incidence in HIV-negative MSM and TGW have varied considerably based upon the country and time in which the study was performed and the population chosen for screening. These rates vary from a longitudinal study in the Netherlands demonstrating no incident HCV infections among HIV-negative MSM over 10,000 person-years (PY) of follow-up^9^ to an incidence of 0.5 cases per 1000 PY in HIV-negative MSM in the U.S.^17^ to an incidence of 1.5 cases per 1000 PY of follow-up in a cohort of HIV-negative MSM receiving services at a sexual health clinic in the United Kingdom (UK).^18^ In this same study, five clusters of genetically linked HCV strains were found using phylogenetic analysis. Using HCV incidence of 1.5 cases per 1000 PY noted in the UK study, one would expect 3 HCV infections among 999 participants tested at month 24 and 1.2 infections among 775 tested at month 12 in the HVTN 505 cohort, more than the 2 HCV incident cases identified.^19^.

Rates of incident HCV in studies of HIV-positive MSM have generally been higher, with a meta-analysis reporting a pooled incidence rate of 6.35 cases per 1000 PY,^3^, though these rates have likely been declining in recent years given the wider availability of HCV treatment.^19^

The relatively low rate of HCV incidence in this population is likely explained by the low rates of IDU (1.3%). As with other populations, IDU is the most important risk factor for HCV acquisition in MSM, and a meta-analysis demonstrated a 34.8% prevalence in this population.^10^ However, it can be difficult to attribute HCV transmission risk to IDU versus risk associated with sexual exposure to blood and other risk factors. Transmission of HCV between sexually linked MSM who deny IDU has clearly been reported in multiple studies.^2,20–24^ Other studies have shown risk factors like sting, use of non-injected drugs before or during sex, anal douching, and concomitant bacterial sexually transmitted infection to be associated with increased HCV transmission risk.^22,25^

Although HBsAb data were not available for all participants, we hypothesize that the low HBV infection rates can be partially attributed to the presumably high HBV vaccination rates in this primarily U.S.-born cohort with a median age of 30. Prior studies have shown higher HBV infection rates in older MSM compared to younger men, correlating inversely with vaccination rates^26,27^ and demonstrated wide variations of prevalence among MSM in the U.S. and across different European countries.^13,27^

Study limitations include the low enrollment of TGW, making it difficult to draw conclusions about this population. Participants were required to meet certain eligibility criteria, including having alanine transaminase (ALT) ≤ 2.5 times upper limit of normal on screening labs and be considered in good general health prior to enrollment, and as such, might not be representative of the population as a whole. Furthermore, individuals volunteering for an HIV vaccine clinical trial may have different attitudes and practices relating to sexual risk, compared to the general population of MSM and TGW. The study did not collect detailed data on specific sexual risk practices that might be associated with non-IDU HCV transmission, such as traumatic sex practices. The high HBV vaccination rates among people born in the U.S. after 1991 make it difficult to apply these findings to older populations, populations in other parts of the world, or evaluate the contribution of tenofovir-containing HIV PrEP to the risk of HBV acquisition and reactivation.

In conclusion, among this population of MSM and TGW enrolled in an HIV vaccine trial, the rates of incident HCV were extremely low, and there were no cases of incident HBV.

## Data Availability

No, I do not have any research data outside the submitted manuscript file.

## Abbreviations

HCV: hepatitis C virus
RNA: HCV ribonucleic acid
HBV: hepatitis B virus
MSM: men who have sex with men
TGW: transgender women
IQR: interquartile range
PrEP: pre-exposure prophylaxis
HVTN: HIV Vaccine Trials Network
HCV Ab: HCV antibody
HBsAb: hepatitis B surface antibody
HBcAb: hepatitis B core antibody
HBsAg: hepatitis B surface antigen
IDU: injection drug use
